# Editorial

**DOI:** 10.2478/v10102-010-0006-2

**Published:** 2010-03-29

**Authors:** Mojmír Mach

**Affiliations:** Institute of Experimental Pharmacology & Toxicology, Dúbravská cesta Bratislava, Slovakia

Modern society is constantly increasing the volume of chemicals for everyday use, including drugs, additives, food stabilizers, cosmetics or ubiquitous by-products of the industry. More than 65,000 chemicals are in the U.S. Environmental Protection Agency's inventory of toxic chemicals and annually the Agency receives approximately 1,500 notices of intent to manufacture new substances. It is thus important to know what each of this compound can do to the living organism.

Thanks to developmental toxicology, it has become evident that even small amounts of potentially toxic compounds introduced to the developing organism may be the cause of later morbidity. This phenomenon is known as developmental origins of health and diseases (DOHaD). It is therefore very important to understand the potential risks.

Toxicology is interdisciplinary science utilizing methods and knowledge from a number of scientific fields like biology, pharmacology, biochemistry, pathophysiology, genetics, etc. Understanding the adverse effects of compounds in the environment may influence our everyday decisions and might shift the course of our life toward happiness.

One of the goals of the Slovak Toxicology Society SETOX, together with the Institute of Experimental Pharmacology & Toxicology SASc., Bratislava, is to impart scientific knowledge and experience to the public, and especially to the young generation. This group has definitely the greatest potential to materialize positive changes in our environment and to promote the protection of our planet. Every experience matters, every encounter, knowledge, situation or person might change our perspective. Thus to pass on knowledge and to educate the young generation is a matter of utmost importance.

There seems to be no safe substance, only its safe use.

